# Efficacy of traditional Chinese exercise for the treatment of pain and disability on knee osteoarthritis patients: a systematic review and meta-analysis of randomized controlled trials

**DOI:** 10.3389/fpubh.2023.1168167

**Published:** 2023-06-08

**Authors:** Shuaipan Zhang, Ruixin Huang, Guangxin Guo, Lingjun Kong, Jianhua Li, Qingguang Zhu, Min Fang

**Affiliations:** ^1^Tuina Department, Yueyang Hospital of Integrated Traditional Chinese and Western Medicine, Shanghai University of Traditional Chinese Medicine, Shanghai, China; ^2^Tuina Department, Shanghai Municipal Hospital of Traditional Chinese Medicine, Shanghai, China; ^3^School of Acupuncture-Moxibustion and Tuina, Shanghai University of Traditional Chinese Medicine, Shanghai, China; ^4^Tuina Department, Institute of Tuina, Shanghai Institute of Traditional Chinese Medicine, Shanghai, China; ^5^Tuina Department, Shuguang Hospital, Shanghai University of Traditional Chinese Medicine, Shanghai, China

**Keywords:** knee osteoarthritis, Traditional Chinese Exercises, WOMAC, systematic review, meta-analysis

## Abstract

**Objective:**

To evaluate the efficacy of Traditional Chinese Exercises (TCEs) in treating knee osteoarthritis (KOA).

**Methods:**

Four databases without language or publication status restrictions were searched until April 1, 2022. Based on the principle of Population, Intervention, Comparison, Outcomes and Study design, the researchers searched for randomized controlled trials of TCEs in treating KOA. The Western Ontario and McMaster Universities Osteoarthritis (WOMAC) pain was defined as the primary outcome, whereas stiffness and physical function were the secondary outcomes. Subsequently, two researchers conducted the process independently, and the data were analyzed using the RevManV.5.3 software.

**Results:**

Overall, 17 randomized trials involving 1174 participants met the inclusion criteria. The synthesized data of TCEs showed a significant improvement in WOMAC pain score [standardized mean difference (SMD) = −0.31; 95% confidence interval (CI): −0.52 to −0.10; *p* = 0.004], stiffness score (SMD = −0.63; 95% CI: −1.01 to −0.25; *p* = 0.001) and physical function score (SMD = −0.38; 95% CI: −0.61 to −0.15; *p* = 0.001) compared with the control group. Sensitivity analyses were performed to determine the combined results' stability, which was unstable after excluding articles with greater heterogeneity. A further subgroup analysis showed that it might be the reason for the heterogeneity of the different traditional exercise intervention methods. Additionally, it showed that the Taijiquan group improved pain (SMD = 0.74; 95% CI: −1.09 to 0.38; *p* < 0.0001; *I*^2^ = 50%), stiffness (SMD = −0.67; 95% CI −1.14 to 0.20; *p* = 0.005) and physical function score (SMD = −0.35; 95% CI: −0.54 to 0.16; *p* = 0.0003; *I*^2^ = 0%) better than the control group. The Baduanjin group improved stiffness (SMD = −1.30; 95% CI: −2.32 to 0.28; *p* = 0.01) and physical function (SMD = −0.52; 95% CI: −0.97 to 0.07; *p* = 0.02) better than the control group. However, the other interventions showed no difference compared with the control group.

**Conclusion:**

This systematic review provides partial evidence of the benefits of TCEs for knee pain and dysfunction. However, due to the heterogeneity of exercise, more high-quality clinical studies should be conducted to verify the efficacy.

**Systematic review registration:**

https://inplasy.com/inplasy-2022-4-0154/, identifier: International Platform of Registered Systematic Review and Meta-analysis Protocols (INPLASY) [INPLSY202240154].

## 1. Introduction

Osteoarthritis (OA) remains the most challenging arthritic disease with a high disease burden and no available disease-modifying treatments. It affects an estimated 240 million people worldwide, including 32 million in the United States (1). Knee osteoarthritis (KOA) is relatively common; a national health survey in the United States (with radiological evidence) shows that 37% of people aged > 60 years have OA, with women accounting for more, and the prevalence continues to increase as the population ages (2). A recent study provides the specific global prevalence [16.0% (95% CI, 14.3–17.8%)] and incidence [203 per 10,000 person-years (95% CI, 106–331)] of KOA (3). It is an obstacle to the repair of stress-induced joint damage because of the abnormal tissue surrounding the knee joint. However, cartilage damage is the most basic pathological feature, and arthritis is also a joint syndrome (4). The symptoms and signs include pain, stiffness, decreased joint movement and muscle weakness. Additionally, long-term adverse outcomes may include limited physical activity, sleep disturbance, fatigue, depression and disability (5). Pain from KOA, which is predictable early in the onset, but becomes unmanageable in the later stages, is difficult to study since it constantly changes. A review showed that pain-related factors include age, gender, obesity, depression and pain sensitization (6). Drugs, including painkillers and anti-inflammatory drugs, have varying degrees of drug resistance and addiction, and high health care costs (5). Therefore, the current treatment strategy has shifted from pharmacologic to non-pharmacologic -based multi-modal combination methods aiming to reduce drug use and increase clinical efficacy. Since the benefits of drugs are limited and evidence has proven that non- pharmacologic therapies are more likely to relieve symptoms in the long term and delay or prevent functional decline, especially exercise has become an important part of KOA treatment, and A guidelines already strongly recommend exercise therapy as the base treatment for knee osteoarthritis pain (7–9). And many high-quality clinical studies have demonstrated the effects of exercise, and a systematic review has shown that it can reduce pain and dysfunction, with the effect lasting for 2–6 months (10). Traditional Chinese exercise (TCE) is also a mind-body exercise used in the management of KOA, which the most familiar type of exercise is Tai Chi. And a trial comparing tai chi and physiotherapy results showed that they had the same improvement effect on the Western Ontario and McMaster Universities Osteoarthritis (WOMAC) questionnaire at 12-week, and greater benefits in the treatment of depression and quality of life (11). Although other traditional exercises such as Baduanjin, Yijinjing, and Wuqinxi have shown evidence of the therapeutic efficacy of KOA, no definite conclusion has been made (12–14). Tai Chi is the most widely applied in the management of chronic diseases of the traditional Chinese exercise, Yijinjing is content elements and shaolin kung fu and traditional Chinese medicine, Wuqinxi is simulation animal behavior way of Chinese traditional sports in ancient China. TCE has similar health benefits to regular exercise therapy, but is characterized by lower energy metabolism as a low—to moderate—intensity physical and mental exercise (15). It emphasizes the coordination and unity of breath and body movements guided by consciousness, while exercising muscles and joints throughout the body (16). Multiple studies have also reported that TCE exercise is thought to relax the body and mind, dilate blood vessels, and promote local blood circulation which may also be a mechanism for pain reduction (17). Therefore, this systematic review and meta-analysis aimed to verify whether TCEs are effective for KOA pain and dysfunction.

## 2. Methods

### 2.1. Registration

This systematic review protocol has been registered on the International Platform of Registered Systematic Review and Meta-analysis Protocols (INPLASY) (registration number: INPLSY202240154) and reported in accordance with the Cochrane Handbook for Systematic Reviews of Interventions (18) and the PRISMA 2020 Checklist (Supplementary material 1).

### 2.2. Inclusion and exclusion criteria for study selection

#### 2.2.1. Types of studies

This study included only randomized controlled trials (RCTs) of TCEs for KOA as the treatment groups. Other designs, such as animal experimentation, case reports and retrospective studies, were excluded.

#### 2.2.2. Types of patients

Patients diagnosed with KOA were included regardless of sex, age, race or severity and disease duration (19). In contrast, patients with KOA associated with severe illnesses such as cancer, liver disease or kidney disease were excluded.

#### 2.2.3. Types of interventions

We included studies using TCEs as the intervention group. For the control group, we included studies using different intervention protocols (i.e., strength exercise, physiotherapy, health education or drug) or those that did not undergo any intervention. However, if other therapies were used in the experimental and control groups, the combination of TCEs and other therapies were included. In addition, there is no limitation to the intervention duration and frequency.

#### 2.2.4. Types of outcome measures

The primary and secondary outcome measures were pain symptoms assessed using the WOMAC and WOMAC stiffness and physical function, respectively (20).

#### 2.2.5. Exclusion criteria

Trials that met any of the following criteria were excluded: (1) quasi-randomized randomized controlled trials and non-randomized trials, (2) duplicated publications, (3) unusable full text or missing data. The two reviewers had resolved all the disagreement with discussion.

### 2.3. Data sources and search strategy

Databases were comprehensively searched from the inception to April 11, 2022, including three English databases [PubMed, Cochrane Central Register of Controlled Trials (CENTRAL) and EMBASE] and one Chinese database [National Knowledge Infrastructure (CNKI)]. Additionally, unpublished resources were collected to ensure the search scope. After discussing with all researchers, a temporary search strategy was identified, comprising the participant, intervention and study design. The search strategies in the PubMed database are shown in Supplementary material 2.

### 2.4. Data collection and analysis

#### 2.4.1. Study selection

**Figure 1:**
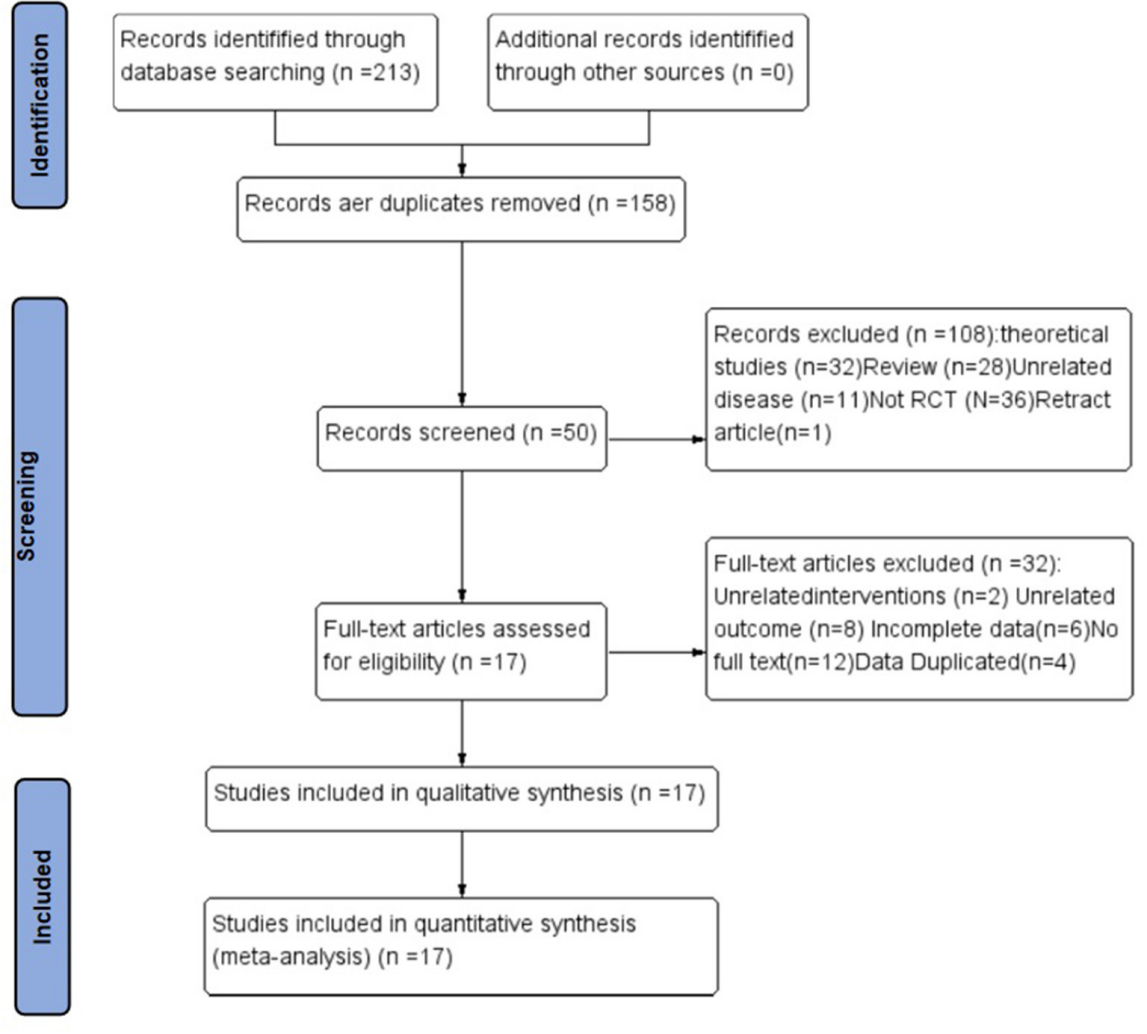
The whole literature selection process. RCT, randomized control trial.

Two researchers (SZ and RH) determined the study selection process according to the previous principle. First, they efficiently screened the duplicates using the literature management system and evaluated the titles and abstracts of the searched studies for eligibility. Subsequently, they assessed the full texts of the remaining studies for final inclusion. Any disagreement on the study selection was resolved through discussion with the third researcher. The study selection process had been reported according to the Preferred Reporting Items for Systematic Reviews and Meta-Analyses guidelines (Figure 1) (21).

#### 2.4.2. Data extraction

Two researchers (GG and JL) independently separated the result information from each investigation according to a standardized data extraction form (Table 1). The extracted data included basic information, participant information, interventions, controls and outcome indicators. The third investigator discussed and negotiated the outcomes when they had different perspectives during the data extraction.

**Table 1:** Characteristics of the included trials and participants.

**Included trials**	**Treatment (duration, frequency)**			**Mean age, y**		**Male, female**		**Sample size (TCEG/CG)**	**BMI Kg/m** ^**2**^	
	**TCEG**	**CG**	**Duration (week)**	**TCEG**	**CG**	**TCEG**	**CG**		**TCEG**	**CG**
Jiang et al. (22) (China)	Baduanjin (5 days weekly, 45 min daily)	Health education (60 min once weekly)	12	64.18 ± 4.09	62.92 ± 5.12			23 (11/12)	25.76 ± 3.41	22.92 ± 2.47
Li et al. (23) (China)	Taijiquan (1 h four times weekly)	Resistance (1 h four times weekly)	16	65.8 ± 6.7	65.9 ± 5.3	19.12	18.12	61 (31/30)	62.6 ± 8.4	61.8 ± 7.9
Fan et al. (24) (China)	Yijinjing (three times weekly for 40 min)	Prokin trial (three times weekly)	8	60.83 ± 9.52	61.80 ± 8.26	5.21	7.19	52 (26/26)	24.96 ± 3.13	24.56 ± 1.19
Zheng et al. (13) (China)	Baduanjin (three sessions weekly lasting 40 min per session)	Not receiving any additional physical training	12	65.11 ± 6.57	63.61 ± 2.63	11.17	8.20	56 (28/28)	24.19 ± 2.37	24.63 ± 2.27
Ye et al. (25) (China)	Baduanjin (three sessions weekly lasting 40 min per session)	Not receiving any additional physical training	12	64.48 ± 7.81	63.08 ± 3.65	12.13	8.17	50 (25/25)	24.15 ± 2.47	24.56 ± 2.31
Brismée et al. (26) (USA)	Taijiquan (featured 6 weeks of group tai chi sessions, 40 min per ession, three times weekly)	Attention control (40-min group meetings three times weekly)	12	70 ± 9.2	68.89 ± 8.9	3.19	4.15	41 (22/19)	27.96 ± 5.92	27.7 ± 6.57
Lee et al. (27) (Korean)	Taijiquan (2 days weekly)	Waiting list control groups	8	70.2 ± 4.8	66.9 ± 6.0	2.27	1.14	44 (29/15)	26.0 ± 3.8	26.0 ± 2.8
Song et al. (28) (Korean)	Taijiquan (at least 3 days weekly)	Routine therapy without participating in any organized exercise program	12	64.8 ± 6.0	62.5 ± 5.6	0.22	0.21	43 (22/21)		
Wang et al. (29) (USA)	Taijiquan (1 h two times weekly)	Wellness education and stretching (1 h two times weekly)	12	63 ± 8.1	68 ± 7.0	4.16	6.14	40 (20/20)	30.0 ± 5.2	29.8 ± 4.3
Wang et al. (11) (USA)	Taijiquan (2 days weekly)	Physical therapy (2 days weekly)	12	60.3 ± 10.5	60.1 ± 10.5	31.75	30.68	204 (106/98)	33.0 ± 7.1	32.6 ± 7.3
Wang et al. (30) (China)	Baduanjin (at least 3 days weekly)	Quadriceps strengthening exercises (at least 3 days weekly)	12	64.74 ± 2.80	65.70 ± 3.50	7,34	8,35	84 (41/43)	23.94 ± 2.02	24.12 ± 2.13
Wortley et al. (31) (USA)	Taijiquan (1 h two times weekly)	resistance training program (1 h two times weekly)	10	68.1 ± 5.3	69.5 ± 6.7	9,3	9,4	25 (12/13)	35.1 ± 5.9	30.5 ± 6.0
Xiao et al. (32) (China)	Wuqinxi (10–15 min of aerobic activity at “rather strenuous level”, Wuqinxi exercise of 40–45 min and cool-down of 5 min)	Physical therapy (4 days weekly)	12	70.7 ± 9.36	70.2 ± 10.35	11.23	12.22	68 (34/34)	27.9 ± 4.75	27.9 ± 4.73
Xiao et al. (12) (China)	Wuqinxi six times weekly, with three groups of exercises each time, and with 5 min' rest between each group of exercises.	No intervention	12	71 ± 2.92	69 ± 3.72	0.132	0.134	266 (132/134)	29.8 ± 7.07	28.4 ± 3.7
Zhang et al. (14) (China)	Yijinjing (40 min two times weekly)	Stretching training exercise (40-min exercise session two times weekly)	12	55.76 ± 8.37	53.40 ± 10.66	4.21	9.16	50 (25/25)	23.5 ± 3.23	22.9 ± 2.98
Zhu et al. (33) (China)	Taijiquan (60 min three times weekly)	Health education (60 min weekly)	24	64.61 ± 3.40	64.53 ± 3.43	0.23	0.23	46 (23/23)	25.23 ± 3.46	25.05 ± 3.42
An et al. (34) (China)	Baduanjin (five times weekly for 30 min)	Not receiving any additional physical training	8	65.4 ± 8.2	64.6 ± 6.7	0.11	0.10	21 (11/10)	25.4 ± 2.9	25.4 ± 2.9

TCEG, Traditional Chinese Exercises Group; CG, Control Group; SD, standard deviation; CI, confidence interval; Std, standardized; df, degrees of freedom; BMI, Body Mass Index.

### 2.5. Assessment of the risk of bias in included studies

Two independent reviewers (RH and QZ) separately assessed the methodological quality using the Cochrane risk of bias tool (35), including the following option: sequence generation; allocation concealment; blinding of participants; blinding of outcome assessment; incomplete outcome data; selective outcome reporting and other issues. Each trial was classified into low, high and ambiguous risks, respectively. Any disagreement was negotiated with the third author to achieve the conclusion.

### 2.6. Data analysis and synthesis

All statistical analyses were performed using Revman version 5.3.0 (Cochrane Collaboration). Quantitative data were extracted from all selected RCTs, including sample size and the mean and standard deviation of outcome measurements post-intervention in each group. Standardized mean difference (SMD) with 95% confidence intervals (CIs) was used to estimate the effect size, and the *I*^2^ test was conducted to evaluate the statistical heterogeneity. If the data had no statistical heterogeneity, a fixed-effect model was used for meta-analysis. However, the source of heterogeneity was further analyzed to exclude the influence of obvious clinical heterogeneity if there was statistical heterogeneity among the results, and a random fixed-effect model was used for the meta-analysis. Heterogeneity in exercise type was investigated using subgroup analysis; sensitivity analyses were performed on the meta-analysis results, which included outcomes for pain, stiffness and physical function using the WOMAC score. Additionally, publication bias was assessed using the illustrations of funnel plots.

## 3. Results

### 3.1. Results of the literature search

Overall, 213 articles were retrieved in **t**his study, of which 55 duplicates were excluded. Additionally, 108 records were excluded after reading the titles and abstracts, including theoretical studies (*n* = 32), traditional reviews (*n* = 28), unrelated diseases (*n* = 11), non-RCT (*n* = 36) and withdrawn articles (*n* = 1). Further literature exclusions (*n* = 32) were performed after reading the full texts of 50 articles with irrelevant interventions (*n* = 2), irrelevant outcomes (*n* = 8), incomplete data (*n* = 6), without full text (*n* = 12) and data duplication (*n* = 4). Finally, 17 articles met the inclusion criteria, and Figure 1 shows the entire literature selection process.

### 3.2. Basic characteristics of the included studies

Two researchers (SZ and RH) independently graded the included papers and extracted data. Overall, 1,174 cases in 17 RCTs were included, of which 598 and 576 were in the treatment and control groups, respectively. The following data were extracted from the retrieved articles: first author, year of publication, study site, patient characteristics, intervention protocol (specific method, duration and frequency, among others) and outcome measures. Table 1 shows the characteristics of the included literature.

### 3.3. Risk of bias in included studies

**Figure 2:**
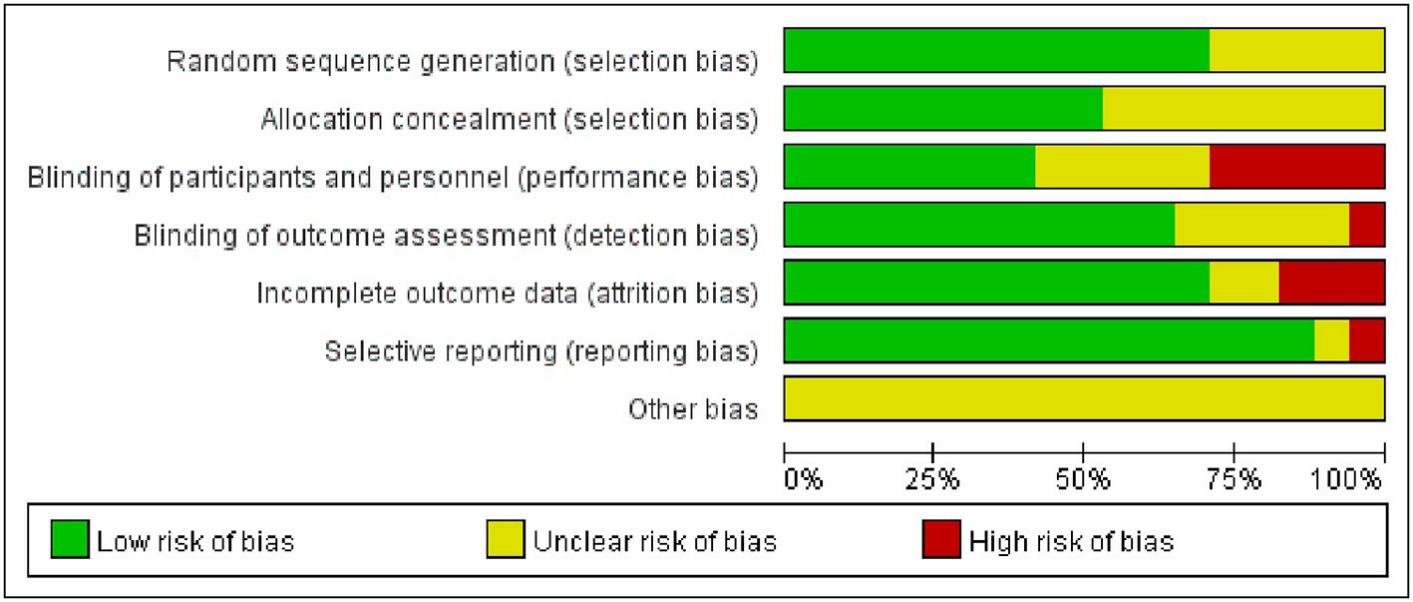
Risk of bias graph: review authors' judgements about each risk of bias item presented as percentages across all included studies.

**Figure 3:**
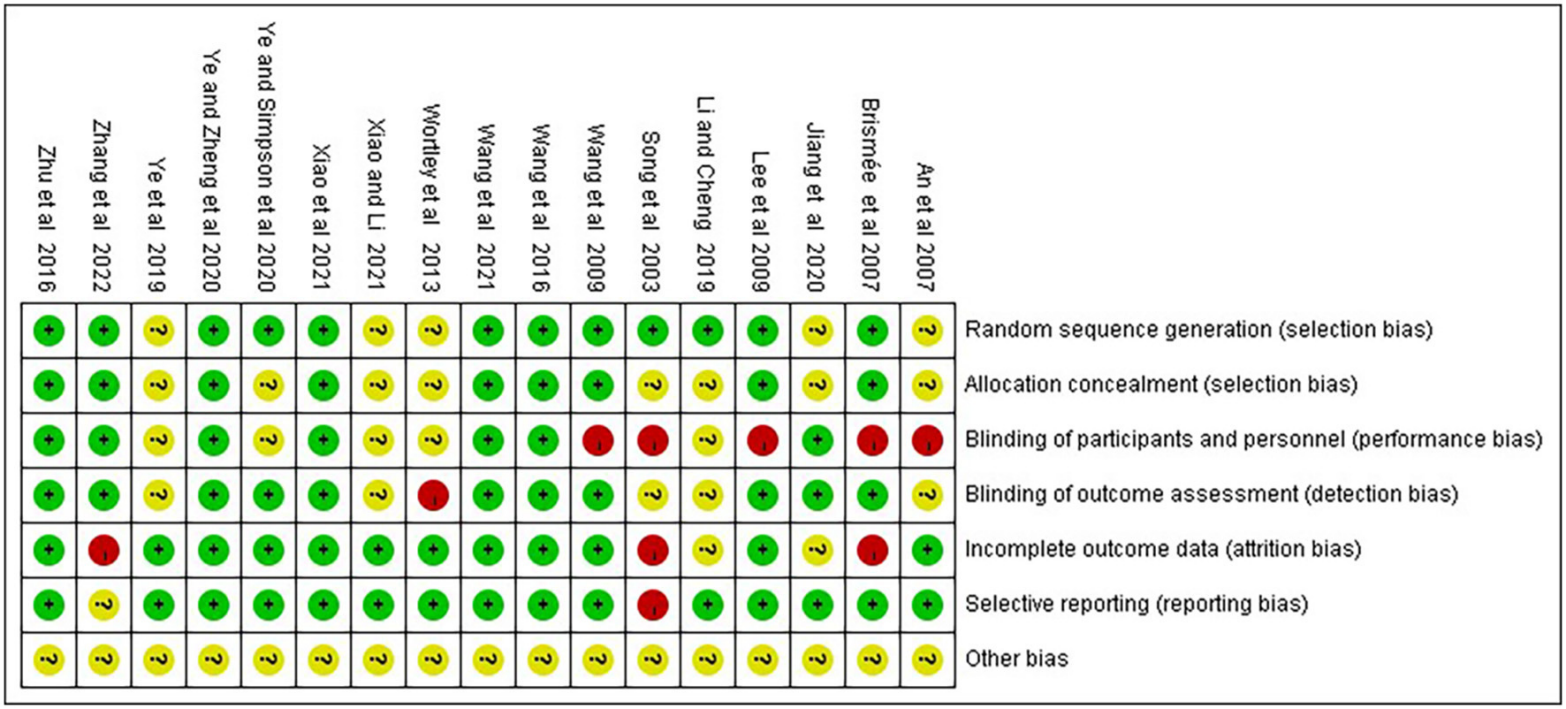
Risk of bias summary: review authors' judgements about each risk of bias item for each included study.

Of the studies that included random sequence generation, 12 were low risk (11–14, 23, 25–30, 33), and five did not clearly describe random sequence generation (22, 24, 31, 32, 34). For allocation concealment risk, nine items (11, 13, 14, 26, 27, 29, 30, 32, 33) were low risk and eight (13, 22, 24, 25, 28, 31, 34) did not clearly describe the method of allocation concealment. Of these studies, only seven had a low risk of performance bias (11, 13, 14, 22, 30, 32, 33). Regarding the risk of measurement bias, 11 studies with blinded outcome assessment were at low risk (11–14, 22, 25–27, 29, 30, 33), one item was high risk (31) and the remaining were not clearly stated (12, 24, 28, 31, 34). Furthermore, most studies were at low risk in terms of risk of follow-up and reporting biases, and only three were at high risk (14, 26, 28). However, the risks of other biases were unknown to all included studies. Figures 2, 3 summarize the results of the risk of bias assessment for all included studies.

### 3.4. Outcome measurements

**Figure 4:**
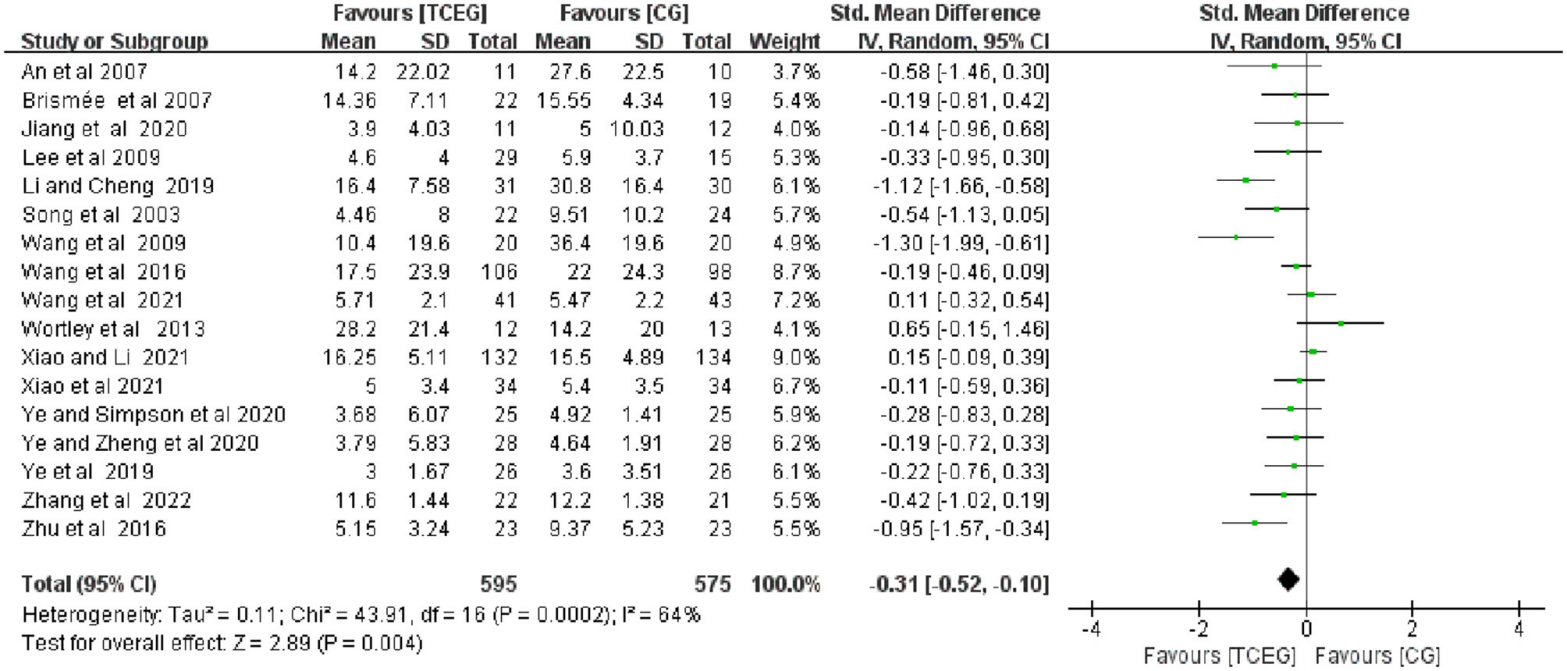
Forest plot of meta-analysis of WOMAC pain score. WOMAC, Western Ontario and McMaster Universities Osteoarthritis Index; TCEG, Traditional Chinese Exercises Group; CG, Control Group; SD, standard deviation; CI, confidence interval; Std, standardized; df, degrees of freedom.

**Figure 5:**
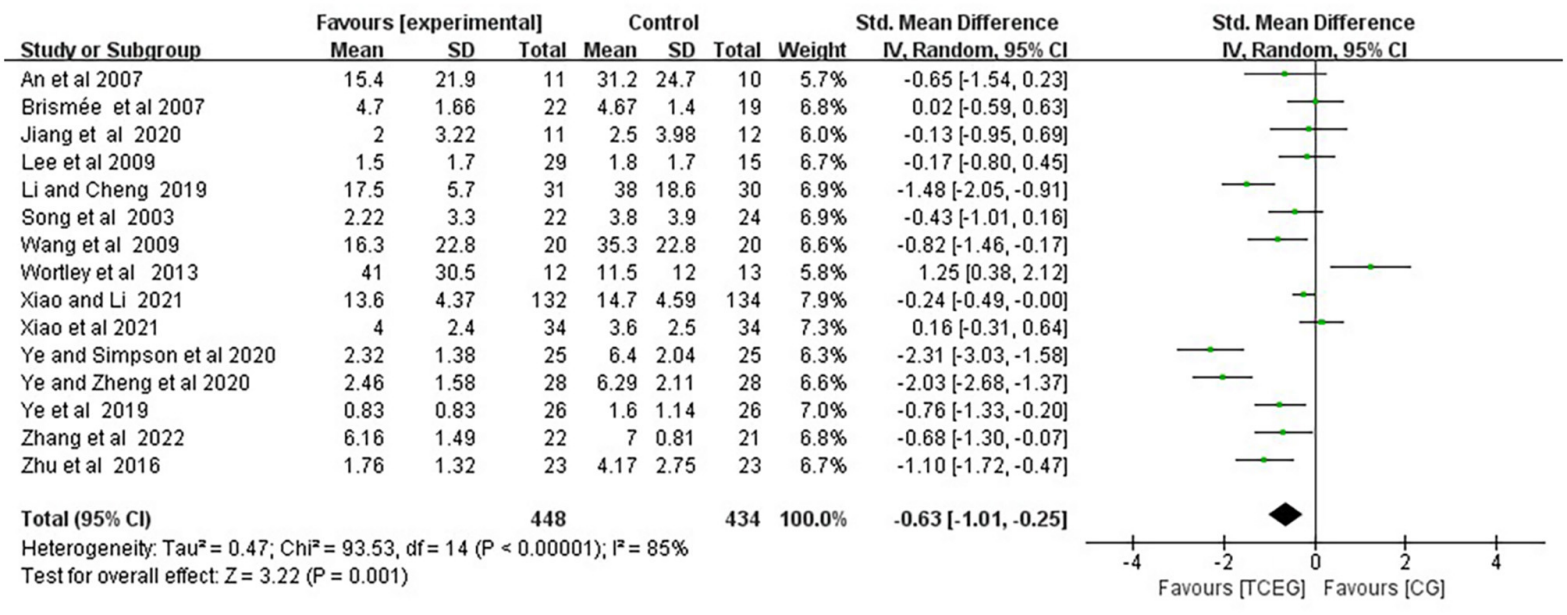
Forest plot of meta-analysis of WOMAC stiffness score. WOMAC, Western Ontario and McMaster Universities Osteoarthritis Index; TCEG, Traditional Chinese Exercises Group; CG, Control Group; SD, standard deviation; CI, confidence interval; Std, standardized; df, degrees of freedom.

**Figure 6:**
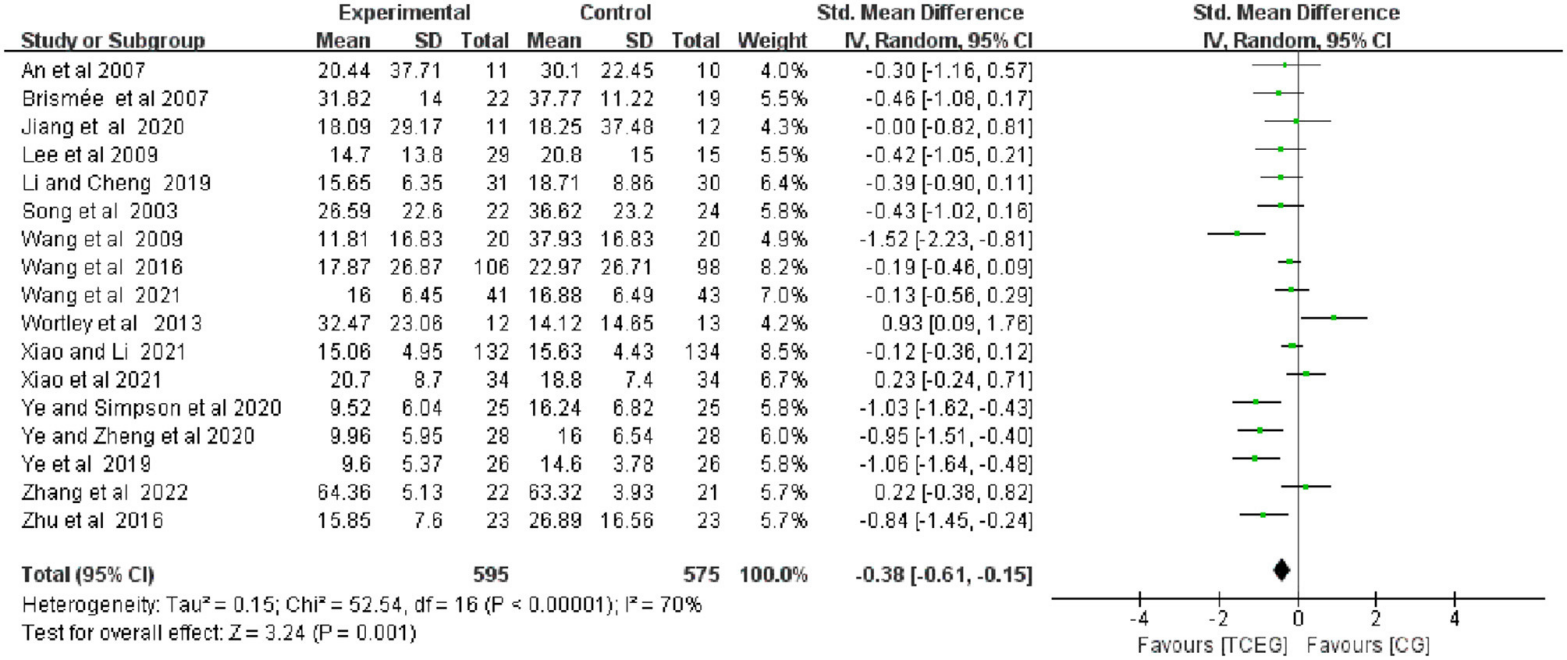
Forest plot of meta-analysis of WOMAC of physical function score. WOMAC, Western Ontario and McMaster Universities Osteoarthritis Index; TCEG, Traditional Chinese Exercises Group; CG, Control Group; SD, standard deviation; CI, confidence interval; Std, standardized; df, degrees of freedom.

The findings of the meta-analyses based on the 17 included studies were presented using the forest plots regarding outcomes of pain, stiffness and physical function using the WOMAC score (Figures 4–6). Because of the significant statistical heterogeneity among the studies, a random fixed-effect model was used for meta-analysis, and the results showed that the synthesized data of TCE group showed a significant improvement in WOMAC pain score (SMD = −0.31; 95% CI: −0.52 to −0.10; *p* = 0.004), stiffness score (SMD = −0.63; 95% CI: −1.01 to −0.25; *p* = 0.001) and physical function score (SMD = −0.38; 95% CI: −0.61 to −0.15; *p* = 0.001) compared with the control group.

The included 17 studies with 1,174 participants (11–14, 22–34) reported the effect of different TCEs on the changes in WOMAC pain score, and the results of a random-effect model meta-analysis showed that after TCE intervention, the improvement in WOMAC pain symptoms (SMD = −0.31; 95% CI: −0.52 to −0.10; *p* = 0.004) was superior to that of the control group, and the heterogeneity of this synthesis was significant (*I*^2^ = 64%). Therefore, these studies used random effects models.

Additionally, the included 15 studies involving 857 participants (11, 13, 14, 22–28, 30, 33, 34) reported the effect of TCE on the changes in WOMAC stiffness, and the results of a random-effect model meta-analysis showed that after TCE intervention, the TCE group effectively relieved the symptoms of WOMAC stiffness (SMD = −0.63; 95% CI: −1.01 to −0.25; *p* = 0.001), and the heterogeneity of this synthetic result was high (*I*^2^ = 85%). Therefore, these studies used random effects models.

The included 17 studies involving 1„174 patients (11–14, 22–34) showed the effect of TCE on WOMAC physical function. Regarding the effect of changes on WOMAC physical function, the results of the random-effects model meta-analysis showed that after TCE intervention, WOMAC Physical function (SMD = −0.38; 95% CI: −0.61 to −0.15; *p* = 0.001) improved better than that of the control group, and there is moderate heterogeneity in improving physical performance. Additionally, this was a combined result (*I*^2^ = 70%). Therefore, these studies used fixed-effects models.

### 3.5. Assessment of reporting bias

**Figure 7:**
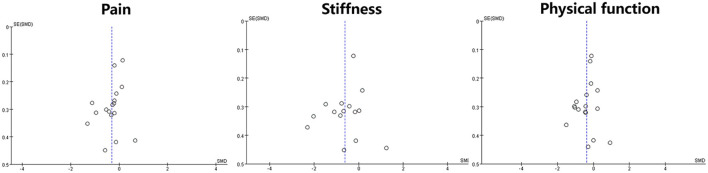
Funnel plot of reporting bias.

A funnel plot of reporting bias was used to compare the WOMAC (pain, stiffness and physical function) scores between the two groups of patients. This study's distribution was asymmetric, suggesting a possible associated reporting bias (Figure 7).

### 3.6. Sensitivity analysis

Given the high heterogeneity and risk of bias, sensitivity analyses were performed to determine the stability of the combined results. After excluding two studies, each associated with pain (12, 23), stiffness (13, 25) and physical function studies (25, 29), the statistical heterogeneity of the combined results showed pain (SMD = −0.29; 95% CI: −0.48 to −0.10; *I*^2^ = 42%), stiffness (SMD = −0.41; 95% CI: −0.72 to −0.10; *I*^2^ = 74%) and physical function (SMD = −0.27, 95% CI: −0.48 to −0.07; *I*^2^ = 59%). Although heterogeneity in the three outcomes was reduced by 22, 11, and 11%, the issue of high heterogeneity still exists, particularly in stiffness. This may be because of the different types of exercise included in the study, the different intervention protocols in the control group (including no intervention, health education, physical therapy, stretching and resistance, among others) and the different duration of the intervention (8–24 weeks), among others.

### 3.7. Subgroup analysis

**Figure 8:**
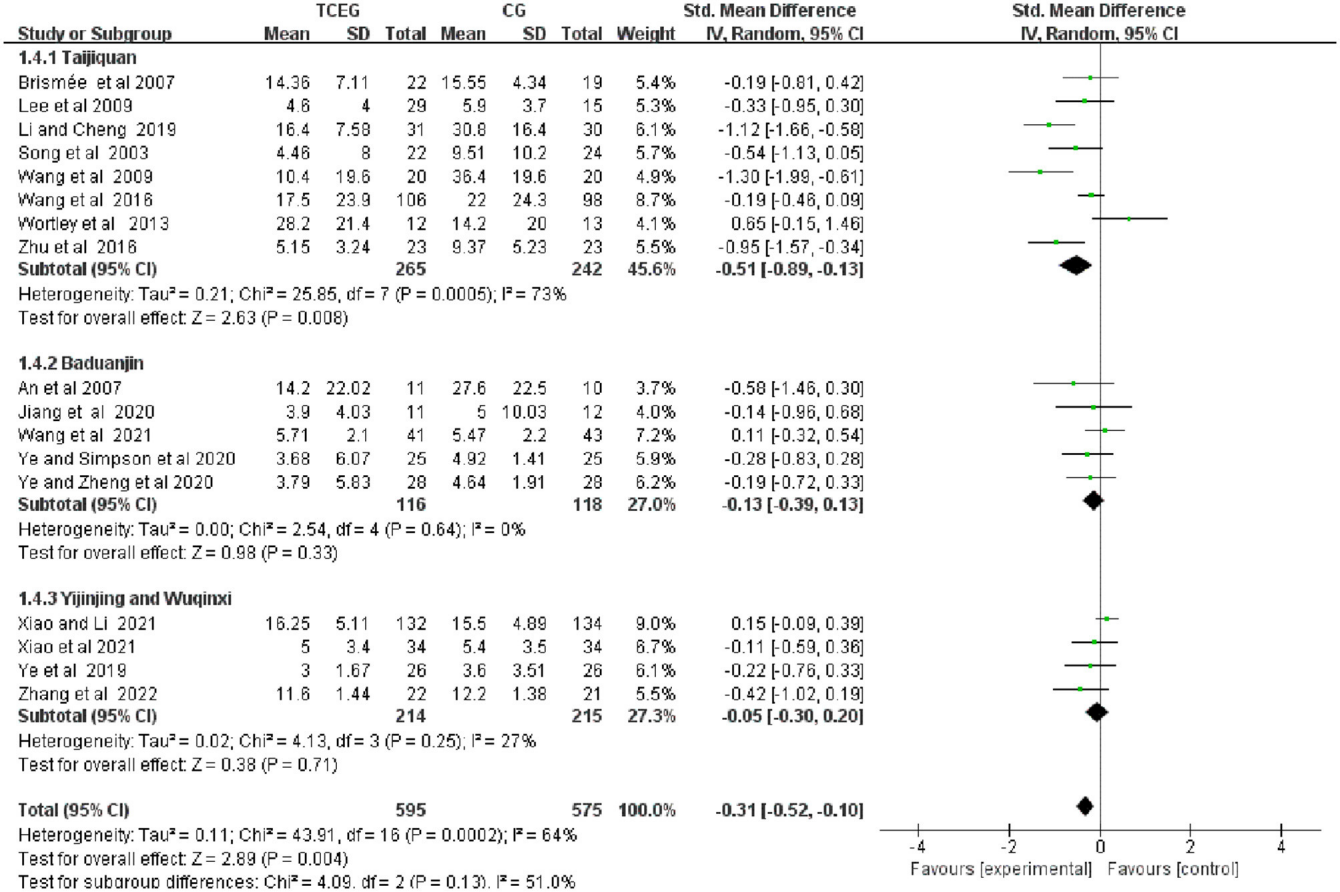
Forest plot of the subgroup analysis in WOMAC pain among the three groups. WOMAC, Western Ontario and McMaster Universities Osteoarthritis Index; TCEG, Traditional Chinese Exercises Group; CG, Control Group; SD, standard deviation; CI, confidence interval; Std, standardized; df, degrees of freedom.

**Figure 9:**
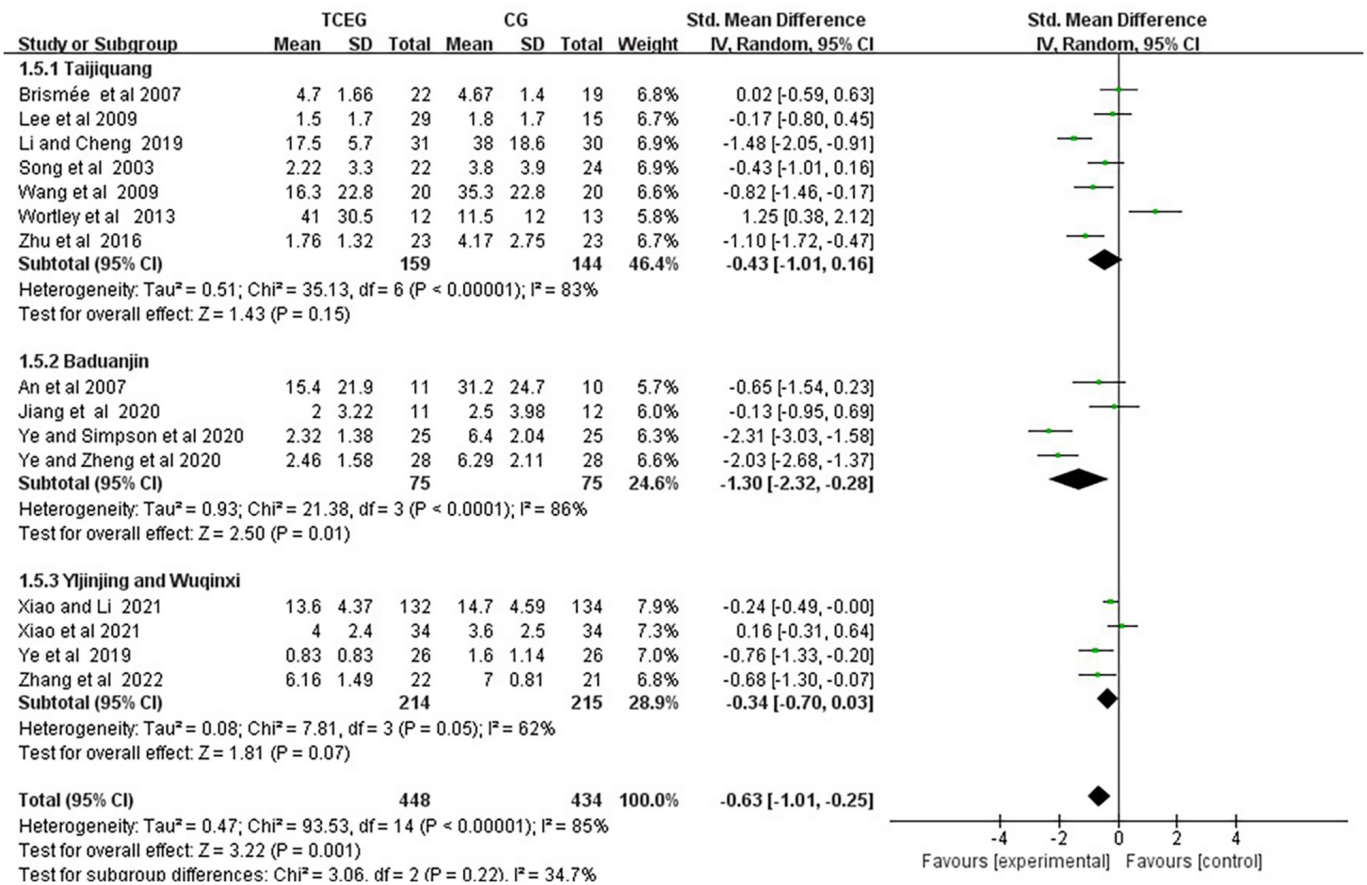
Forest plot of the subgroup analysis in WOMAC stiffness among the three groups. WOMAC, Western Ontario and McMaster Universities Osteoarthritis Index; TCEG, Traditional Chinese Exercises Group; CG, Control Group; SD, standard deviation; CI, confidence interval; Std, standardized; df, degrees of freedom.

**Figure 10:**
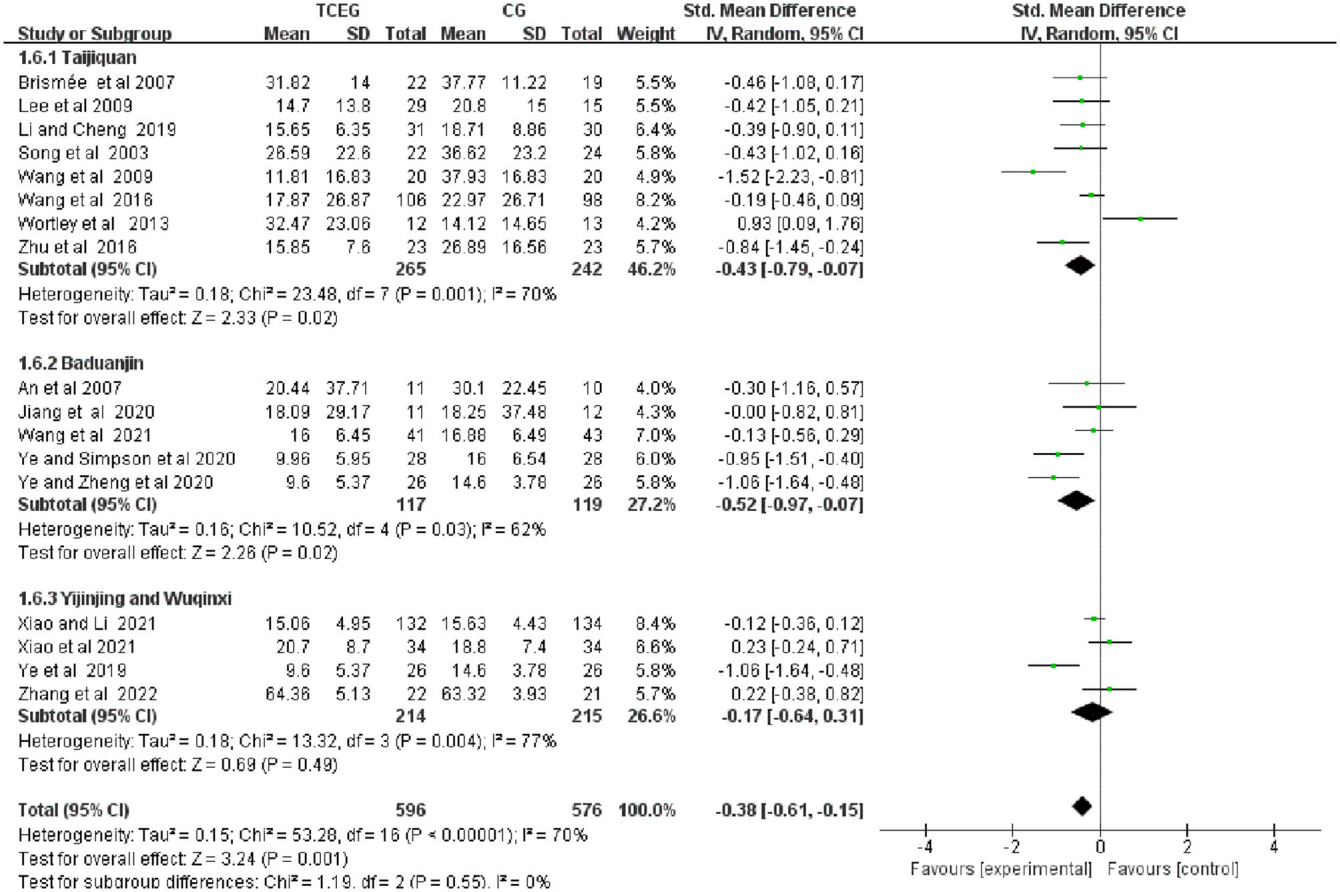
Forest plot of the subgroup analysis in WOMAC physical function among the three groups. WOMAC, Western Ontario and McMaster Universities Osteoarthritis Index; TCEG, Traditional Chinese Exercises Group; CG, Control Group; SD, standard deviation; CI, confidence interval; Std, standardized; df, degrees of freedom; BMI, Body Mass Index.

Subgroup analysis was performed according to the exercise types of the 17 studies, which were classified into three groups as follows: the Taijiquan group, the Baduanjin group and the other groups. Figures 8–10 present all relevant data for subgroup meta-analyses.

Subgroup analysis showed a significant improvement in pain scores in the Taijiquan group (SMD = −0.51; 95% CI: −0.89 to 0.13; *p* = 0.008) compared with the control group. However, the heterogeneity in the Taijiquan group was still greater *I*^2^ = 73%, and the pain score after excluding two studies by Wang et al. and Wortley et al. (8, 18) from the Taijiquan group was *I*^2^ = 50% (SMD = −0.74; 95% CI: −1.09 to 0.38; *p* < 0.0001). The high heterogeneity in this part may be because of the significant risk of bias in these two items. A limitation of Wang et al.'s study is that patients were unblinded to their treatment group assignment. However, the Wortley et al. study was pseudo-randomized, depending on the sex and pain scores of Westerners. Pain scores in the Baduanjin group (SMD = −0.13; 95% CI: −0.39 to 0.13; *p* = 0.33) and the other groups (SMD = −0.05; 95% CI: −0.30 to 0.20; *p* = 0.71) suggest that the two groups of TCEs had no difference in the pain symptoms of KOA compared with the control group.

The result showed that the stiffness symptom scores of the Taijiquan group (SMD = −0.43; 95% CI: −1.01 to 0.16; *p* = 0.15) and the Yijinjing and Wuqinxi groups (SMD = −0.34; 95 % CI: −0.70 to 0.03; *p* = 0.07) were not different from that of the control group; the stiffness score in the Baduanjin group (SMD = −1.30; 95% CI: −2.32 to 0.28; *p* = 0.01) was better than that in the control group. Additionally, the Taijiquan group found a significant difference (SMD = −0.67, 95% CI: −1.14 to 0.20, *p* = 0.005) after excluding the study by Wortley et al. (31). The possible reason is that the risk of inclusion in this study was high. According to the conclusion of the original text, the WOMAC stiffness score of the Taijiquan group was barely improved and had a moderate impact on the WOMAC physical function score.

As shown in Figure 10, the physical function scores in the Taijiquan (SMD = −0.43; 95% CI: −0.79 to 0.07; *p* = 0.02) and Baduanjin (SMD = −0.52; 95% CI −0.97 to 0.07; *p* = 0.02) groups were better than that in the control group. Additionally, the physical function in the Yijinjing and Wuqinxi groups (SMD = −0.17; 95% CI: −0.64 to 0.31; *p* = 0.49) compared with the control group had no significant difference. However, after excluding two studies (29, 31) because of the high heterogeneity of the Taijiquan group, the group's physical function score was better improved (SMD = −0.35; 95% CI: −0.54 to 0.16; *p* = 0.0003; *I*^2^ = 0%).

## 4. Discussion

This meta-analysis showed that TCEs are associated with the efficacy of WOMAC (pain, stiffness and physical function) in patients with KOA, which was consistent with the included article results section of the study (11–14, 22, 24, 25, 27, 30, 33). However, due to higher heterogeneity and risk bias, sensitivity analyses were used to check the stability of results by reducing the heterogeneity by omitting two studies per group, which did not significantly alter these results. These results were generally consistent regardless of TCEs duration and frequency. Subgroup analysis showed that only Taijiquan improved pain symptoms compared with the control group regarding the primary outcome measure of WOMAC pain score. However, analysis of the secondary outcomes showed that Taijiquan and Baduanjin improved stiffness symptoms better than the control group, and Taijiquan improved physical function better than the control group, while Yijinjing and Wuqinxi showed no advantages in improving pain, stiffness and physical function symptoms. Therefore, these findings can provide patients with more precise adjuvant treatment plans.

The analysis was performed due to the difference in the clinical application of traditional exercise therapy. Taijiquan, Baduanjin, Yijinjing and Wuqinxi were kinds of physical and mental exercise that combine the characteristics of meditation and physical exercise (9, 14, 36, 37). Baduanjin was a simpler mind-body exercise that may be easier for older adults, enabling them to focus more on breathing and controlling their movements (38). Although two Taijiquan groups mentioned symptoms of discomfort in the included articles, one (26) had mild muscle soreness and complaints of foot and knee pain in the first few days, and one (29) complained of pain in the feet and knees. Participants reported increased knee pain at the 2-week assessment, which resolved after modifying the participants' Taijiquan technique. Additionally, three cases (14) were disqualified due to worsening symptoms in the study on Yijinjing, and the author did not mention the specific reasons. Therefore, the detailed mechanisms of TCEs were closely associated with the curative effect, and trainers with certain experience were needed to apply them better. Although limited by insufficient included studies, particularly Yijinjing and Wuqinxi, the two exercises should be combined into one group for subgroup analysis. Further relevant research is urgently needed to analyse the difference between the two for a more precise application. Additionally, the limitations of the literature should be verified by more rigorously designed and high-quality clinical studies in the future.

Although the underlying mechanism of TCEs for KOA was unclear, it may be an effective treatment option for patients. For relevant studies, publication bias should be awarded in the included RCTs and whether there were significant gaps in methodological characteristics. Additionally, this study found that half of the included RCTs had a sample size < 50, some lacked allocation concealment and subjective and reporting bias, and TCEs interventions differed in the type of exercise and duration (4–24 weeks). There were differences in frequency and recipient populations. Consequently, we cannot decide on the type of exercise to choose, quantify its duration and frequency or make the best training recommendations for those who need it. Several studies have investigated the efficacy of TCEs for KOA, not as monotherapy but as adjunctive therapy; however, it was difficult to determine whether TCEs were used alone or as a synergistic intervention. Some researchers found that the incidence of KOA is positively correlated with body weight and age. Therefore, the higher their age and body weight, the higher the incidence rate they will encounter (24, 25). Although this study collected related data, the difference was not obvious.

This review is an evaluation of the curative effect of TCEs on pain dysfunction of KOA. Similar reviews have been published before, and this study is an update of previous reviews. In addition, although the conclusion of the whole review cannot give the effect of TCEs on KOA, it also introduces the status quo of different types of TCEs intervention on KOA for public. The differences between different exercise are also analyzed, which may be a feature of this article.

## 5. Conclusion

This systematic review provides partial evidence of the benefits of TCEs for knee pain and dysfunction. However, the results were unstable, which may be because of the heterogeneity of the exercise intervention methods; therefore, more high-quality clinical studies should be conducted to verify the efficacy of TCEs intervention on patients with KOA.

## Data availability statement

The original contributions presented in the study are included in the article/Supplementary material, further inquiries can be directed to the corresponding authors.

## Author contributions

SZ, RH, QZ, and MF conserved, designed the study, and wrote the draft manuscript. RH, SZ, GG, and JL performed the literature search and extracted data. MF and QZ assessed the methodological quality. LK, QZ, and MF performed data synthesis and analysis. All authors were involved in interpreting the data, contributed to the final manuscript, and read and approved the final manuscript.
