# Supplementary material for: Efficacy of traditional Chinese exercise for the treatment of pain and disability on knee osteoarthritis patients: a systematic review and meta-analysis of randomized controlled trials
Source: Front Public Health. 2023 Jun 8;11:1168167. doi: 10.3389/fpubh.2023.1168167 (PMC10285305; doi:10.3389/fpubh.2023.1168167)
Supplement: Supplementary file 1 [file Data_Sheet_1.docx]

Supplementary file. The search strategies in PubMed database.

**#1 Intervention:** (("Qigong"[Mesh]) OR ((((((((((((((((Qi Gong[Title/Abstract]) OR (Ch'i Kung[Title/Abstract])) OR (Traditional Chinese exercises[Title/Abstract])) OR (daoyin[Title/Abstract])) OR (gongfa[Title/Abstract])) OR (Taijiquan[Title/Abstract])) OR (Tai Chi[Title/Abstract])) OR (Tai-ji[Title/Abstract])) OR (Chi, Tai[Title/Abstract])) OR (Ji Quan, Tai[Title/Abstract])) OR (Quan, Tai Ji[Title/Abstract])) OR (Tai Chi Chuan[Title/Abstract])) OR (Wuqinxi[Title/Abstract])) OR (Baduanjin[Title/Abstract])) OR (Yijinjing[Title/Abstract])) OR (Liuzijue[Title/Abstract]))).

**#2 Participant:** ("Osteoarthritis, Knee"[Mesh]) AND ((((((((Knee Osteoarthritis) OR Knee Osteoarthritis) OR Osteoarthritis, Knee) OR Osteoarthritis Of Knee) OR Knee, Osteoarthritis Of) OR Knees, Osteoarthritis Of) OR Osteoarthritis Of Knees) OR Knee, pain)OR Knee ,disorder))

**#3 Study design:** (randomized controlled trial [pt] OR controlled clinical trial [pt] OR randomized [tiab] OR placebo [tiab] OR clinical trials as topic [mesh: noexp] OR randomly [tiab] OR trial [ti]) NOT (animals [mh] NOT humans [mh])

**#4** #1 AND #2 AND #3
